# BMP Stimulation Differentially Affects Phosphorylation and Protein Stability of β-Catenin in Breast Cancer Cell Lines

**DOI:** 10.3390/ijms25094593

**Published:** 2024-04-23

**Authors:** Mustafa Ilhan, Nurcan Hastar, Branka Kampfrath, Deniz Neslihan Spierling, Jerome Jatzlau, Petra Knaus

**Affiliations:** 1 Institute of Chemistry and Biochemistry, Freie Universität Berlin, 14195 Berlin, Germany; mustafa.ilhan@fu-berlin.de (M.I.); nurcan.hastar@fu-berlin.de (N.H.); branka.kampfrath@fu-berlin.de (B.K.); denizspierling@gmail.com (D.N.S.);; 2 Berlin School of Integrative Oncology, Charité—Universitätsmedizin Berlin, 13353 Berlin, Germany; 3 Brandenburg School for Regenerative Therapies, Charité—Universitätsmedizin Berlin, 13353 Berlin, Germany

**Keywords:** WNT, BMP, crosstalk, β-CATENIN, breast cancer

## Abstract

Increased expression and nuclear translocation of β-CATENIN is frequently observed in breast cancer, and it correlates with poor prognosis. Current treatment strategies targeting β-CATENIN are not as efficient as desired. Therefore, detailed understanding of β-CATENIN regulation is crucial. Bone morphogenetic proteins (BMP) and Wingless/Integrated (WNT) pathway crosstalk is well-studied for many cancer types including colorectal cancer, whereas it is still poorly understood for breast cancer. Analysis of breast cancer patient data revealed that *BMP2* and *BMP6* were significantly downregulated in tumors. Since mutation frequency in genes enhancing β-CATENIN protein stability is relatively low in breast cancer, we aimed to investigate whether decreased BMP ligand expression could contribute to a high protein level of β-CATENIN in breast cancer cells. We demonstrated that downstream of BMP stimulation, SMAD4 is required to reduce β-CATENIN protein stability through the phosphorylation in MCF7 and T47D cells. Consequently, BMP stimulation reduces β-CATENIN levels and prevents its nuclear translocation and target gene expression in MCF7 cells. Conversely, BMP stimulation has no effect on β-CATENIN phosphorylation or stability in MDA-MB-231 and MDA-MB-468 cells. Likewise, SMAD4 modulation does not alter the response of those cells, indicating that SMAD4 alone is insufficient for BMP-induced β-CATENIN phosphorylation. While our data suggest that considering BMP activity may serve as a prognostic marker for understanding β-CATENIN accumulation risk, further investigation is needed to elucidate the differential responsiveness of breast cancer cell lines.

## 1. Introduction

Bone morphogenetic proteins (BMP) and Wingless/Integrated (WNT) pathways are two evolutionarily conserved, core signaling cascades playing crucial roles in development and tissue homeostasis as well as in the formation of cancer [1,2]. BMPs are the largest subgroup of the transforming growth factor-β (TGF-β)/BMP family of secreted growth factors, which initiate signaling upon binding and recruitment of two types of transmembrane serine/threonine kinase receptors into a hetero-tetrameric complex composed of two type I and two type II receptors. To activate the pathway, ligands can either bind to a preformed hetero-tetrameric complex consisting of type I (e.g., ALK3/BMPR1a or ALK6/BMPR1B) and type II (e.g., BMPR2) receptors, or binding of a ligand to the type I receptors may trigger the formation of a BMP-induced signaling receptor complex [3,4]. Within the ligand-bound complex, the constitutively active type II receptor phosphorylates and activates the type I receptor at the glycine–serine-rich GS-domain, and thereby initiates the phosphorylation and activation of receptor-regulated SMADs (R-SMADs). Activated R-SMADs interact with the co-mediator SMAD, SMAD4, to drive nuclear translocation of the SMAD complex and to initiate transcriptional regulation of SMAD-target genes. However, BMP receptors could also signal through non-SMAD pathways, activating other pathways such as PI3K/Akt and MAPK [5]. Likewise, the WNT pathway consists of β-CATENIN dependent (canonical) and independent (non-canonical) signaling paths [6]. β-CATENIN is the core mediator of the canonical WNT pathway. Therefore, the amount of β-CATENIN in the cell is tightly regulated at the localization, protein stability and transcriptional levels. In the absence of a WNT ligand, β-CATENIN is constitutively phosphorylated and is targeted for ubiquitin-mediated proteasomal degradation by β-CATENIN-destruction complex, which consists of three main proteins: AXIN1, casein kinase-1 (CK1) and glycogen synthase kinase-3β (GSK3β) [7]. Priming phosphorylation of β-CATENIN by CK-1 at Ser 45 is followed by GSK3β phosphorylation at Ser 33, 37 and Thr 41 [7,8]; thereby, protein stability of β-CATENIN decreases and is targeted for ubiquitination-mediated proteasomal degradation. Binding of WNT ligands to the Frizzled (FZ) receptor and co-receptor, such as low-density lipoprotein-related protein 5/6 (LRP5/6), triggers the phosphorylation and dimerization of the receptor–coreceptor complex. Once activated, the complex interacts with Disheveled (DVL) and in turn recruits the destruction complex to the proximity of the membrane, keeping β-CATENIN in the unphosphorylated state with increased protein stability. Thus, β-CATENIN accumulates in the cytoplasm, followed by nuclear translocation, where it interacts with other transcription factors, such as T Cell-Factor/Lymphoid-Enhancer Factor (TCF/LEF) to regulate the transcription of target genes such as *c-MYC* and *AXIN2* [9,10].

During early embryogenesis, BMPs are important mediators of fate determination in myogenic progenitor cells, where they commit precursor cells to osteoblastic differentiation. The cooperative work of the BMP and WNT pathways was previously shown for osteoblast differentiation [11,12,13,14], and they enhance the activity of each other through the regulation of core mediators at RNA and/or protein level [15,16] in murine cell lines. On the other hand, they may also counteract the effect of each other during their crosstalk in other contexts. During crypt formation, a high level of BMP pathway activity suppresses β-CATENIN [17,18] and thereby defines the region for villi in the intestine [19]. Likewise, BMPs suppress WNT-induced proliferation in favor of differentiation and facilitates ventralization during the patterning in mice [20], chicken [21], and Xenopus [22] embryos. Further, expression of the BMP inhibitor, BAMBI, leads to increased nuclear translocation of β-CATENIN in the C2C12 murine myoblast cell line [23], and it enhances the WNT/β-CATENIN signal activity in adipogenesis [24]. Overall, these findings suggest that the mode of their interaction is tissue- and context-dependent in development and homeostasis. In addition to their evident crosstalk in tissue homeostasis, the imbalanced interaction of the two pathways may also contribute to the initiation and progression of carcinogenesis. Sun et al. (2021) demonstrated that the WNT agonist roof plate-specific spondin-2 (RSPO2) triggers endocytosis and degradation of the BMP type I receptor, BMPR1A, in acute myeloid leukemia cell lines [25]. Likewise, Farrell et al. (2012) demonstrated that stimulation with BMP4 counteracts the mutant β-CATENIN-induced cancer stem cell properties and enhances differentiation in the spheroid model of mouse intestine [26]. On the contrary, isocitrate dehydrogenase 1 (IDH-1) mutant gliomas have higher expression of BMP4, and it is required for the upregulation of the WNT pathway to enhance the tumor aggressiveness in human glioblastoma cell lines [27]. The same cooperative interaction has been shown in the context of prostate cancer, where BMP activity could contribute to bone metastasis downstream of the WNT pathway [28]. In addition, bone stromal cell-derived WNT5a stimulates BMP6 expression in prostate cancer cell lines and reverses the suppression of hormone therapy-mediated cell proliferation of cancer cells in vitro [29].

Breast cancer is the most diagnosed type of cancer and the leading cause of cancer-related death among women worldwide [30]. Although it is mostly curable when it is diagnosed in the early stages, the chance of treatment decreases with advanced stages of cancer progression, mainly due to metastasis [31]. Therefore, it is vital to elaborate on the underlying mechanism and potential contributors of disease progression for better treatment approaches. Aberrant activity of WNT signaling and/or accumulation of β-CATENIN in the cytoplasm and in the nucleus have been frequently seen in breast cancer, playing a critical role in breast cancer metastasis [32,33,34]. However, inhibition strategies for targeting β-CATENIN are not effective enough as an option for treatment so far [35,36]. High mutation rates in β-CATENIN and/or WNT-related genes are major drivers for increased levels of β-CATENIN in colorectal carcinoma and hepatocellular carcinoma, whereas the mutation frequency in genes encoding the respective proteins is quite rare or not present in breast cancer [37]. Instead, overexpression of WNT ligands or silencing of WNT antagonists is responsible for increased β-CATENIN levels in breast cancer [38,39]. Although the interaction between the BMP and WNT pathways is well studied for other types of cancer, particularly in colorectal cancer, their interaction in breast cancer is still poorly understood. In this study, we aimed to investigate the molecular details of BMP and WNT crosstalk in the context of breast cancer. To demonstrate this, we focused on differentially expressed BMP ligands in breast cancer patients. Human breast cancer cell lines were stimulated with BMP2 and/or BMP6 to examine whether reversing the reduced BMP ligand expression could have an impact on β-CATENIN levels. 

In the context of breast cancer, our results revealed that BMPs reduce β-CATENIN protein stability through direct involvement of SMAD4 in MCF7 and T47D cells; in turn, SMAD4 affects the nuclear accumulation and target gene expression of β-CATENIN in MCF7 cells. However, our observation in a comparative dataset on two other breast cancer lines suggests that BMP-induced regulation of β-CATENIN may require the contribution of additional factors other than SMAD4 and thus occurs in a cell-dependent manner. 

## 2. Results

### 2.1. BMP Stimulation Triggers Phosphorylation and Proteasomal Degradation of β-CATENIN in MCF7 Cells

**Figure 1:**
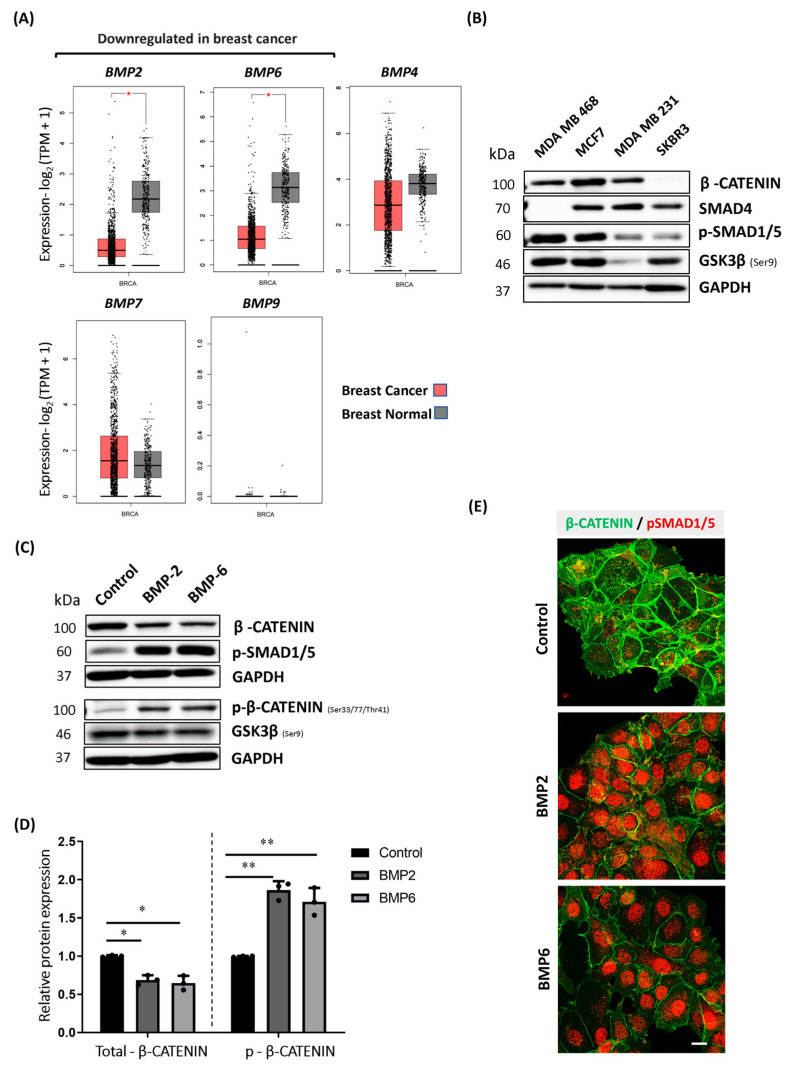
BMP stimulation triggers phosphorylation and proteasomal degradation of β-CATENIN in MCF7 cells. (**A**) Analysis of TCGA breast tissue samples. Comparison of mRNA expression levels of selected BMPs between tumor tissue from breast cancer patients (TCGA dataset) (*n* = 1085) and available data from TCGA normal tissue and Genotype-Tissue Expression (GTEx) (*n* = 291) in the GEPIA2 database. The method for differential analysis is one-way ANOVA, using disease state (Tumor or Normal) as a variable for calculating differential expression (*p* < 0.01). Default settings were used, and analysis was retrieved by GEPIA2 web server (Tang, Z. et al., 2017 [42]) (**B**) Endogenous protein expression level of total β-CATENIN, SMAD4, phosphorylated SMAD1/5 and GSK3β (Ser9) in human breast cancer cell line panel. GAPDH was shown as a loading control for the comparison of protein expression levels. (**C**) Representative Western blot images showing protein levels of phosphorylated SMAD1/5, total and phosphorylated β-CATENIN and phosphorylated GSK3β at Ser9 (inactive) upon BMP stimulation (10 nM BMP2 or BMP6) in MCF7. Cells were starved for 3 h and stimulated with BMPs in growth medium for 24 h. (**D**) Densitometric analysis of respective protein levels in total cell lysates. GAPDH was used as an equal loading control, and all values were normalized to GAPDH prior to relative protein expression analysis. Data are represented as the mean + SD of three independent experiments (* *p* < 0.05, ** *p* <0.01). (**E**) Confocal microscopy imaging of MCF7 cells after 24 h of BMP stimulation (10 nM BMP2 or BMP6) (Scale bar = 10 μm).

Based on reported evidence that the BMP pathway suppresses canonical WNT pathway activity during development and inhibits the progression of several types of cancer, e.g., colorectal cancer [40], we aimed to investigate the effect of BMPs on WNT signaling in the context of breast cancer cells. BMPs have both tumorigenic and tumor-suppressive roles in breast cancer [41]. Therefore, our first step was to screen and analyze the differential mRNA expression profiles of all available BMP ligands in TCGA breast cancer patient datasets covered in the GEPIA2 database [42]. TCGA data analysis demonstrated that mRNA expression of *BMP2* and *BMP6* are significantly downregulated in breast cancer patients when compared to normal breast tissue (Figure 1A). Consistently, Liu et al. reported that reduced *BMP2* and *BMP6* levels are correlated with reduced overall and relapse-free survival rates in breast cancer [43]. On the other hand, the *BMP9* mRNA level is almost undetectable, and the expression of *BMP4* and *BMP7* expression is comparable to that of healthy counterparts (Figure 1A). Furthermore, we demonstrated that the downregulation of *BMP2* and *BMP6* is not tumor-subtype-specific, and the same expression pattern is present across different molecular subtypes (Supplementary Figure S1A,B). This implies that the downregulation of *BMP2* and *BMP6* might affect the survival and progression of breast cancer regardless of molecular subtype.

We analyzed the β-CATENIN protein expression levels in the human breast cancer cell line panel (Figure 1B). Since we detected the highest expression level of total β-CATENIN in MCF7 cells (Figure 1B, second lane in the upper row), we first activated the BMP pathway through BMP2 and BMP6 stimulation in MCF7 cells to test the effect on the β-CATENIN protein level. Both BMP2 and BMP6 stimulation significantly reduced (~30%) the protein level of total β-CATENIN upon 24 h stimulation (Figure 1C, upper panel and Figure 1D, left panel), which was also evident upon BMP4 stimulation (Supplementary Figure S1C, left panel). However, neither BMP2 nor BMP6 affected the expression of β-CATENIN at the mRNA level (Supplementary Figure S1D). We observed a similar effect of decreased total β-CATENIN staining after BMP2/6 stimulation using confocal microscopy (Figure 1E). Since the protein stability of β-CATENIN is crucial for the canonical WNT pathway, we next asked whether the phosphorylation status of β-CATENIN is affected upon BMP stimulation. Consistent with the decrease in total β-CATENIN protein level, both BMP2 and BMP6 stimulation significantly increased (~2-fold) the phosphorylation of β-CATENIN at Ser 33, 37 and Thr 41 residues, indicative for subsequent proteasomal degradation (Figure 1C, lower panel, and Figure 1D, right panel). Further, we showed that BMP stimulation triggers a decrease in total β-CATENIN level in a dose-dependent manner (Supplementary Figure S1C, right panel). Consistently, the level of Ser9-phosphorylated inactive GSK3β, which is known to phosphorylate the respective residues to target for proteasomal degradation [8], decreases upon BMP stimulation (Figure 1C, lower panel and Supplementary Figure S1E). These observations suggest that BMP stimulation may negatively regulate WNT signaling by affecting β-CATENIN protein stability in MCF7 cells.

### 2.2. BMP-Induced Phosphorylation of β-CATENIN Requires BMP Type I Receptor Kinase Activity and SMAD4 in MCF7 Cells

**Figure 2:**
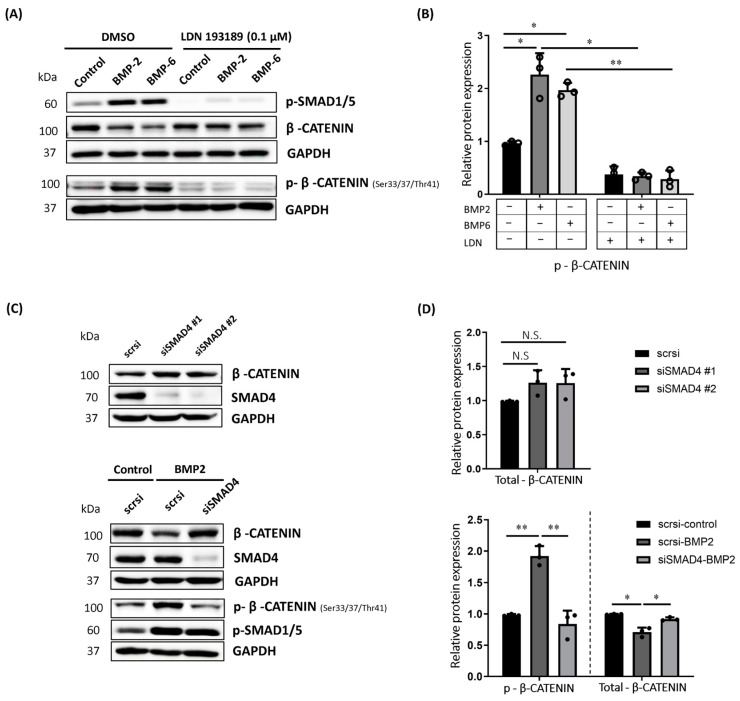
BMP-induced degradation of β-CATENIN requires BMP type I receptor kinase activity and SMAD4 in MCF7 cells. (**A**) Representative Western blot images showing protein levels of phosphorylated SMAD1/5, total and phosphorylated β-CATENIN in MCF7 cells. LDN193189 treatment inhibited BMP type I receptor activity and BMP-induced phosphorylation of β-CATENIN at Serine 33,37 and Threonine 41 residues in 24 h. Prior to BMP stimulation, cells were treated with 100 nM of LDN193189 for 3 h during starvation, then stimulated with BMPs for 24 h. (**B**) Densitometric analysis of protein level of phosphorylated β-CATENIN at Serine 33,37 and Threonine 41 residues in total cell lysates. All values were normalized to GAPDH prior to relative protein expression analysis. Statistical significance was calculated by two-way ANOVA. (**C**) Protein levels of SMAD4 and total β-CATENIN after siRNA-mediated silencing of SMAD4 with two different siRNAs (upper panel, “#” stands for the number of siRNA) and effect of SMAD4 silencing on BMP-induced regulation of total and phosphorylated β-CATENIN protein level in MCF7 cells (lower panel). After transfection, cells were starved for 3 h, followed by 24 h of BMP2 (10 nM) stimulation. (**D**) Densitometric analysis of respective protein levels in total cell lysates. GAPDH was used as an equal loading control, and all values were normalized to GAPDH prior to relative protein expression analysis. Data are represented as the mean + SD of three independent experiments (N.S: Not significant, * *p* < 0.05, ** *p* < 0.01).

Since BMPs signal either through SMAD or non-SMAD signaling pathways, our next aim was to understand which path triggers the phosphorylation and reduced protein stability of β-CATENIN. To clarify this, we first inhibited the kinase activity of the BMP type I receptor by using the chemical inhibitor LDN193189 targeting type I receptors [44,45]. The LDN193189 concentration and the duration of treatment were decided according to previous publications [46,47]. As expected, LDN193189 treatment diminished BMP-induced phosphorylation of SMAD1/5 (Figure 2A, upper panel). Notably, LDN193189-mediated inhibition of kinase activity of BMP type I receptors significantly impaired the BMP-induced phosphorylation of β-CATENIN at Ser 33, 37 and Thr 41 residues (Figure 2A lower panel, Figure 2B). Consistently, BMP-induced decrease in total β-CATENIN was reversed in the presence of LDN193189 compared to the LD193189-treated control group (Figure 2A, upper panel).

This was the first hint that BMP-induced phosphorylation of β-CATENIN to target for degradation is regulated through the involvement of the BMP type I receptor directly. However, LDN193189 is not a BMP type I receptor-specific small molecule inhibitor and could also affect other targets [46]. Of note, some of those targets of LDN193189 involve downstream mediators of the non-SMAD signaling pathway. Therefore, we speculated that LDN could inhibit some other kinases, which, in turn, could lead to a misinterpretation of the results. To exclude this scenario, we followed a complementary approach, and inhibited the expression of SMAD4 by using siRNA-mediated silencing in MCF7 cells. If the decrease we observed was regulated via a SMAD-dependent pathway, we would rescue the decreased protein stability of β-CATENIN in the absence of SMAD4. We performed silencing of SMAD4 by using two different siRNAs and we successfully reduced endogenous SMAD4 level to different extents in MCF7 cells (Figure 2C, upper panel) (Supplementary Figure S2A). Of note, the knockdown of SMAD4 increased the total β-CATENIN (~30%) at the protein level in the absence of BMP stimulation, supporting our hypothesis for the involvement of SMAD4 in the regulation of β-CATENIN level (Figure 2C,D, upper panel). Remarkably, SMAD4 deficiency impaired the BMP-induced phosphorylation of β-CATENIN at the respective residues (Figure 2C, lower panel and Figure 2D, lower panel). Furthermore, siRNA-mediated inhibition of SMAD4 almost completely reversed the BMP-induced decrease in total β-CATENIN protein level (Figure 2C,D, lower panel) (Supplementary Figure S2B). With this, we confirmed that BMP-induced change in the β-CATENIN protein phosphorylation results from BMP type I receptor-mediated and SMAD4-dependent activation of the BMP pathway in MCF7 cells.

### 2.3. BMP Stimulation Reduced the Cytoplasmic Accumulation and Nuclear Translocation of β-CATENIN in MCF7 Cells

**Figure 3:**
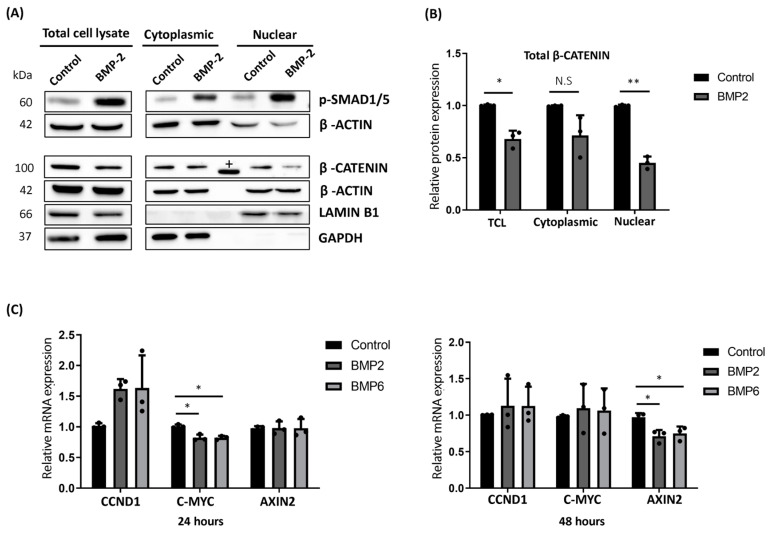
BMP stimulation reduced the cytoplasmic accumulation and nuclear translocation of β-CATENIN in MCF7 cells. (**A**) Representative Western blot images showing protein levels of phosphorylated SMAD1/5 and total β-CATENIN upon BMP2 (10 nM) stimulation for 48 h in different cellular fractions of MCF7 cells. LAMIN B1 and GAPDH were used as positive and negative control for nuclear fraction, respectively. (For cytoplasmic fraction vice versa.) (+: This band belongs to the protein ladder between cytoplasmic and nuclear fractions.) (**B**) Densitometric analysis of total β-CATENIN protein levels in total cell lysates (TCL), cytoplasmic and nuclear fraction of MCF7 cells, respectively. Prior to relative protein quantification, samples were normalized to β-ACTIN as equal loading controls. (**C**) Relative mRNA expressions of WNT target genes; *CCND1*, *C-MYC*, and *AXIN2* were analyzed by qRT-PCR after 24 h or 48 h of BMP2 or BMP6 (10 nM) stimulation in MCF7 cells. Cells were starved for 3 h, followed by BMP stimulation for respective time points. All targets were normalized to *GAPDH* as a housekeeping gene prior to relative expression quantification. Data are represented as the mean + SD of three independent experiments (N.S: not significant, * *p* < 0.05, ** *p* < 0.01, unless otherwise stated, comparisons were N.S).

β-CATENIN acts as a co-transcription factor, and upon its nuclear translocation, it directly regulates the expression of several oncogenic genes including *CCND1* and *c-MYC* at the transcript level [48]. Since β-CATENIN accumulation in cytoplasm and increased nuclear translocation are considered as poor prognostic markers and are frequently observed in most of the malignancies [49,50,51], we performed cell fractionation to assess the change in subcellular localization of β-CATENIN upon BMP2 stimulation in MCF7 cells. Consistently, BMP stimulation reduced the β-CATENIN level in total cell lysates (TCL) (Figure 3A,B, left panel). 

Along with decreased protein levels of β-CATENIN in TCL, nuclear translocation of β-CATENIN was significantly reduced (~50%) upon BMP stimulation in MCF7 cells (Figure 3A,B, right panel). We observed a similar decreasing pattern for the cytoplasmic fraction of total β-CATENIN (Figure 3A,B, middle panel). Next, we wanted to understand the effect of BMP stimulation on the expression of β-CATENIN target genes following BMP stimulation by using quantitative PCR (qPCR). As expected, the BMP target genes, *ID1* and *ID2*, were upregulated after 24 and 48 h of stimulation (Supplementary Figure S3). Furthermore, 24 h of BMP stimulation reduced the mRNA expression of *c-MYC* (~20%) (Figure 3C, left panel), whereas a significant reduction (~30%) was only observed for *AXIN2* at the end of 48 h of BMP stimulation (Figure 3C, right panel). Intriguingly, BMP stimulation transiently increased the mRNA level of *CCND1* after 24 h of stimulation (Figure 3C, left panel), which was not observed at the end of 48 h (Figure 3C, right panel). Altogether, our expression data showed that BMP-induced degradation of β-CATENIN has a functional impact on nuclear translocation and was partially mirrored in the expression of target gene expression, implying a possible compensation mechanism through the involvement of other interacting pathways.

### 2.4. BMP-Induced Degradation of β-CATENIN Alters E-CADHERIN Localization in MCF7 Cells

**Figure 4:**
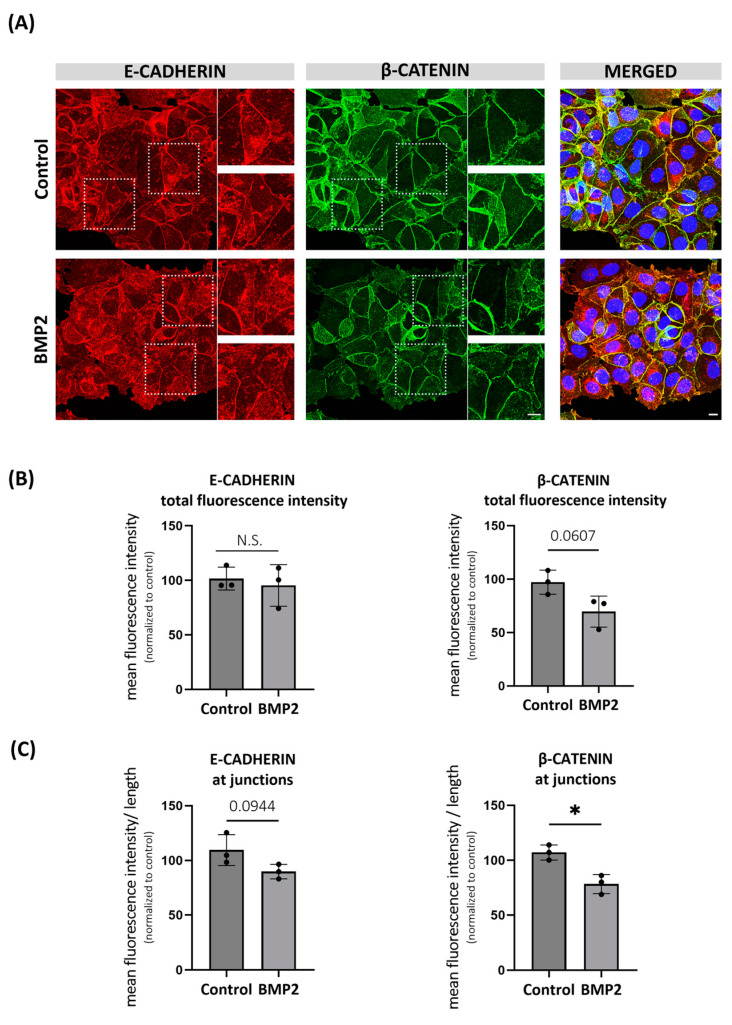
BMP-induced degradation of β-CATENIN partially disrupts E-CADHERIN localization in MCF7 cells. (**A**) Representative confocal microscopy images showing the localization and fluorescence intensity of E-CADHERIN and total β-CATENIN after 24 h of BMP2 (10 nM) stimulation in MCF7 cells. Prior to stimulation, cells were starved for 3 h. Scale bar = 10 μm. Selected areas were shown to indicate the change in localization of E-CADHERIN with reduced junctional β-CATENIN level. (**B**) Quantification of total fluorescence intensity of E-CADHERIN and total β-CATENIN upon BMP stimulation. (**C**) Quantification of fluorescence intensity of E-CADHERIN and total β-CATENIN at junctions. Semi-automated ImageJ macro JunctionMapper was used for the quantification (Brezovjakova H. et al., 2019 [57]). Data are represented as the mean + SD of three independent experiments. (N.S: not significant, * *p* < 0.05).

BMPs have been reported to possess a dual role in epithelial-to-mesenchymal transition (EMT) and migration of various types of cancer. For instance, BMP2 was shown to have both pro- and anti-migratory effects in human breast cancer cell lines [52,53]. Although β-CATENIN is mainly known for its role as a co-transcription factor, it was first identified as a cytoplasmic component of the junctional complexes [54], where it interacts with the cytoplasmic domain of E-CADHERIN and links to the actin cytoskeleton [55]. Therefore, we asked whether E-CADHERIN expression and/or localization is disrupted by BMP-induced destabilization of β-CATENIN and if it affects the cellular migration in MCF7 cells. As expected, we observed a decreasing trend in total β-CATENIN signal intensity upon BMP2 stimulation (Figure 4B, right panel). Further, we showed that β-CATENIN mainly localizes on the plasma membrane of MCF7 cells (Figure 4A, middle panel), and its junctional localization was significantly reduced by BMP2 stimulation (Figure 4C, right panel). Likewise, we observed that BMP2 stimulation caused a decreasing trend for the junctional localization of E-CADHERIN (Figure 4A,C, left panel), whereas the total protein intensity of E-CADHERIN remained unchanged (Figure 4B, left panel), suggesting that reduced β-CATENIN might partially trigger the internalization of E-CADHERIN. 

We performed wound healing assays to investigate a possible correlation between disruption in E-CADHERIN membrane localization and cellular migration. Contrary to previous findings [53,56], BMP2 stimulation did not trigger the migration of MCF7 cells in our study (Supplementary Figure S4A,B). Instead, BMP2 stimulation caused a decreasing trend in the migration ability of MCF7 cells at 18 and 26 h of the assay, and this was partially reversed by silencing of SMAD4 (Supplementary Figure S4A–C). These observations suggest that partial disruption of E-CADHERIN membrane localization might not be enough to trigger cellular migration.

### 2.5. BMP Stimulation Differentially Affects β-CATENIN Protein Level in MDA MB 468, T47D and MDA MB 231 Cells

**Figure 5:**
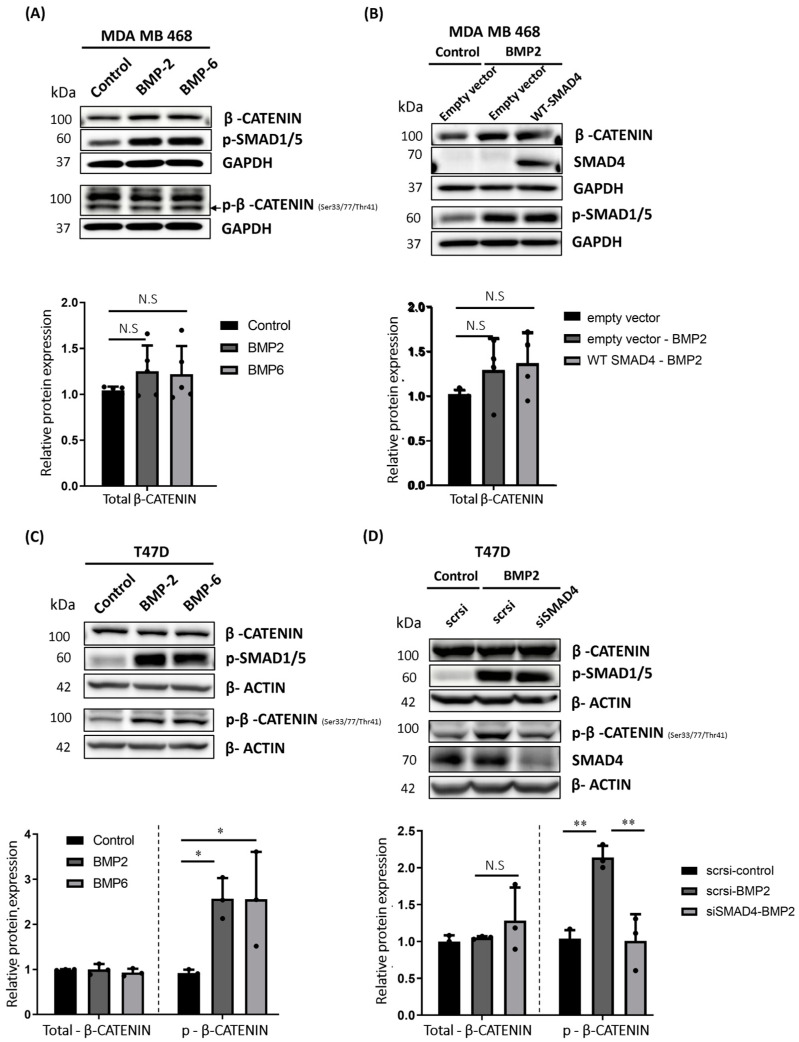
BMP stimulation differentially affects β-CATENIN protein stability in MDA MB 468 and T47D cells. (**A**) Representative Western blot images showing total and phosphorylated β-CATENIN, phosphorylated SMAD1/5 and densitometric analysis of total β-CATENIN in MDA MB 468 cells after 24 h of BMP2 or BMP6 stimulation (10 nM). Cells were starved for 3 h and stimulated with BMPs for 24 h. GAPDH was used as an equal loading control, and all values were normalized to loading control prior to relative protein expression analysis. (**B**) Representative Western blot images showing total β-CATENIN, SMAD4 (IB: FLAG), phosphorylated SMAD1/5 and densitometric analysis of total β-CATENIN in MDA MB 468 cells. Cells were transfected with FLAG-tagged human wild-type SMAD4 construct. At 24 h post-transfection, cells were starved for 3 h, followed by 24 h of BMP2 (10 nM) stimulation. GAPDH was used as an equal loading control, and all values were normalized to loading control prior to relative protein expression analysis. (**C**) Representative Western blot images showing total and phosphorylated β-CATENIN, phosphorylated SMAD1/5 and densitometric analysis of total and phosphorylated β-CATENIN in T47D cells after BMP2 or BMP6 stimulation (10 nM). Cells were starved for 3 h and stimulated with respective BMPs for 24 h. β-ACTIN was used as an equal loading control, and all values were normalized to loading control prior to relative protein expression analysis. (**D**) Representative Western blot images of total and phosphorylated β-CATENIN, SMAD4, phosphorylated SMAD1/5 and densitometric analysis of total and phosphorylated β-CATENIN in T47D cells. After silencing SMAD4, cells were starved for 3 h and stimulated for an additional 24 h with BMP2 (10 nM). β-ACTIN was used as an equal loading control, and all values were normalized to loading control prior to relative protein expression analysis. Data are represented as the mean + SD of at least three independent experiments. (N.S: not significant, * *p* < 0.05, ** *p* < 0.01).

Having found that SMAD4 is required for BMP-induced phosphorylation and degradation of β-CATENIN in MCF7 cells, we wondered whether we could extend our observation to other breast cancer cell lines. To do this, we followed a complementary approach, where we selected a SMAD4-negative human breast cancer cell line [58], MDA MB 468, and aimed to determine the effect of BMP stimulation on β-CATENIN protein stability in the absence of endogenous SMAD4 expression. Opposing MCF7 cells, BMP stimulation of MDA MB 468 cells did not trigger the phosphorylation of β-CATENIN (Figure 5A, upper panel), which is required for targeting β-CATENIN to proteasomal degradation. Instead, our data suggest that BMP2 or BMP6 stimulation could cause an increasing trend in the total protein level of β-CATENIN in MDA MB 468 (Figure 5A, lower panel). Next, we expressed FLAG-tagged human SMAD4 in MDA MB 468 cells and did not observe an impact on β-CATENIN protein level (Figure 5B). To analyze another SMAD4-positive breast cancer cell line, we repeated the experiments in MDA MB 231 cells (Figure 1B). Similarly to MDA MB 468 cells, BMP stimulation neither decreased the total protein level nor affected the phosphorylation of β-CATENIN at respective residues (Supplementary Figure S5A). In addition, siRNA-mediated silencing of SMAD4 did not have any impact on the total protein level of β-CATENIN (Supplementary Figure S5B) in MDA MB 231 cells.

MDA MB 231 and MDA MB 468 cell lines represent the triple negative breast cancer (TNBC) molecular subtype, whereas the MCF7 cell line represents the luminal molecular subtype of breast cancer. Therefore, we wanted to understand whether this could affect the outcome of BMP stimulation for the regulation of β-CATENIN protein stability. To test this, we next used the T47D human breast cancer cell line, which represents the same molecular subtypes and is similar to the features of MCF7 cells. Consistent with our observation in MCF7 cells, BMP2 and/or BMP6 stimulation significantly induced the phosphorylation of β-CATENIN at respective residues, required to target β-CATENIN for the ubiquitination-mediated proteasomal degradation (Figure 5C, upper and Figure 5C, lower right panel). On the other hand, we did not observe a change in the total protein level β-CATENIN (Figure 5C, upper panel, and Figure 5C, lower left panel). Furthermore, BMP-induced phosphorylation of β-CATENIN was inhibited by siRNA-mediated silencing of SMAD4 (Figure 5D, upper panel and Figure 5D, lower right panel), whereas the total protein level of β-CATENIN was not affected by BMP stimulation (Figure 5D, upper panel, and Figure 5D, lower right panel). Our data in T47D cells further confirmed that BMPs induce the phosphorylation of β-CATENIN in a SMAD4-dependent manner. On the other hand, when we consider the overall results in the latter three cell lines, it also suggests that BMP-induced regulation of β-CATENIN protein stability is not straightforward, and we cannot generalize to all breast cancer cell lines. This regulation may occur in a cell line-specific manner.

### 2.6. TGF-β Pathway Suppression Contributes to the Regulation of β-CATENIN Protein Stability in MCF7 Cells

The BMP and TGF-β pathways are known to balance the activity of each other, particularly during development. Besides, it was already reported that SMAD3 is a binding partner for β-CATENIN to protect it from proteasomal degradation [59], and SMAD3-mediated nuclear translocation of β-CATENIN is required for TGF-β target gene activation [60].

**Figure 6:**
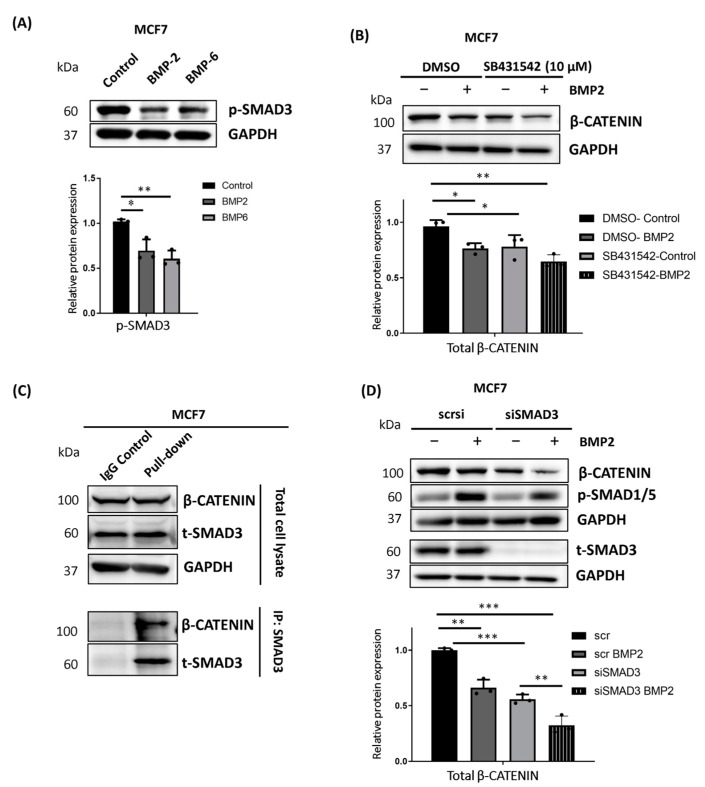
β-CATENIN interacts with SMAD3 in MCF7 cells. (**A**) Representative Western blot images showing the change in the protein level of p-SMAD3 and its densitometric analysis upon BMP2 or BMP6 stimulation (each 10 nM). Cells were starved for 3 h, followed by 24 h of BMP stimulation. GAPDH was used as equal loading control, and all values were normalized to loading control prior to relative protein expression analysis. (**B**) Representative Western blot images and densitometric analysis showing the change in total β-CATENIN protein level after SB431542-induced chemical inhibition of TGF-β pathway activity in the absence or presence of BMP2 stimulation. During starvation, cells were treated with 10 μM SB431542 or an equal volume of DMSO for 3 h. Then, cells were stimulated by 10 nM BMP2 for 24 h. GAPDH was used as equal loading control, and all values were normalized to loading control prior to relative protein expression analysis. (**C**) Co-immunoprecipitation assay showing the interaction of total SMAD3 with β-CATENIN in MCF7 cells. GAPDH was used as housekeeper. (**D**) Representative Western blot images showing the effect of siRNA-mediated silencing of SMAD3 on total β-CATENIN, p-SMAD1/5 and total SMAD3 proteins and densitometric analysis showing the change in total β-CATENIN protein level in MCF7 cells. After 24 h of transfection, cells were starved for 3 h and stimulated for an additional 24 h with BMP2 (10 nM). All values were normalized to a loading control prior to relative protein expression analysis. All quantified data are represented as the mean + SD of three independent experiments. Two-way analysis of variance (ANOVA) with post hoc Tukey HSD test was used for SMAD3 silencing and SB431542 experiments. (* *p* < 0.05, ** *p* < 0.01, *** *p* < 0.001).

Therefore, we questioned whether BMP pathway activation has a different impact on TGF-β signaling in MCF7 and MDA MB 231 cell lines. BMP stimulation only suppressed the TGF-β pathway activity in MCF7 cells (Figure 6A, upper panel), which was evident by the significant decrease in phosphorylated-SMAD3 (p-SMAD3) protein level upon BMP stimulation (Figure 6A, lower panel). However, p-SMAD3 protein level was not affected in MDA MB 231 cells upon BMP pathway activation (Supplementary Figure S6A), indicating that this might be the reason for the differential response of those cell lines to BMP-induced β-CATENIN regulation. We confirmed that SMAD3 and β-CATENIN interact with each other in both MCF7 (Figure 6C) and MDA MB 231 cells (Supplementary Figure S6B). To test this, we either silenced SMAD3 or chemically inhibited the kinase activity of the TGF-β type I receptor by using SB431542, which was shown as a selective inhibitor for the TGF-β type I receptor [61]. We showed that siRNA-mediated silencing of SMAD3 or SB431542-mediated inhibition of TGF-β activity were enough to reduce the total β-CATENIN protein level even without BMP stimulation in MCF7 cells. Furthermore, SMAD3 depletion or chemical inhibition of the TGF-β pathway enhanced the effect of BMP stimulation on the regulation of β-CATENIN protein level (Figure 6B,D). This supports that BMP-induced suppression of the TGF-β pathway activity might contribute to the BMP-mediated regulation of β-CATENIN. On the other hand, neither SMAD3 silencing nor chemical inhibition of TGF-β pathway activity reduced the total β-CATENIN protein level in the absence or presence of BMP stimulation in MDA MB 231 cells (Supplementary Figure S6C,D). Although our data suggest that the suppression of TGF-β pathway activity may contribute to the BMP-induced regulation of β-CATENIN, it also implies that BMP-induced suppression of the TGF-β pathway might not be the major trigger, and it rather enhances the effectiveness of the BMP-induced phosphorylation and the regulation of β-CATENIN protein stability.

Taken together, our findings provided evidence that BMP stimulation induces phosphorylation, which regulates the protein stability of β-CATENIN in a SMAD4-dependent manner in MCF7 and T47D cells. On the other hand, we observed different responses upon BMP stimulation in other human breast cancer cell lines, which suggests that SMAD4 may not be enough, and the critical involvement of other mediators is needed for the responsiveness to this regulation in breast cancer cell lines. Depending on the genetic background of the cancer cells as well as the combination and/or availability of other mediators, cells may lose the ability to respond to BMP-induced regulation of β-CATENIN in a cell- and context-dependent manner. Therefore, it is important to further understand the reason behind the differential response of cell lines before considering BMP pathway activity as a potential player for regulating β-CATENIN protein level in breast cancer patients.

## 3. Discussion

BMP and WNT signaling pathways may either promote or inhibit the activity of each other in a tissue- and context-dependent manner [14,26]. Increased nuclear translocation of β-CATENIN has been frequently seen in breast cancer [34,35]. However, current treatment strategies used to reduce β-CATENIN protein stability and its nuclear accumulation are not as efficient as desired [36]. Therefore, it is important to reveal the mediators and mechanism of their crosstalk to improve our understanding. β-CATENIN protein stability is mainly mediated by the suppression of WNT inhibitors and/or the upregulation of WNT activators’ expression in breast cancer [38,39]. Consistently, epigenetic silencing of genes encoding for secreted WNT inhibitors such as *SFRP1*, *SFRP2* and *SFRP5* was previously reported for the cell lines we used here [62]. Notably, all these cell lines express at least one of the canonical WNT ligands, which suggests that they may activate the WNT pathway in an autocrine/paracrine manner [62,63]. These findings also imply that human breast cancer cell lines may retain the same mechanism for the elevated level of β-CATENIN in cell culture. In this study, we aimed to investigate the impact of BMP stimulation on the canonical WNT pathway by focusing on the regulation of β-CATENIN protein stability. We here demonstrated, for the first time, that increased BMP activity triggers phosphorylation of β-CATENIN and reduces its protein stability, i.e., thereby targeting β-CATENIN for degradation. This (i) is regulated through the kinase activity of the BMP type I receptor and (ii) requires the direct involvement of SMAD4 in MCF7 human breast cancer cell line, potentially through the suppression of TGF-β signaling, indicating that the previously described crosstalk between BMP and WNT pathways [25,26,27,28,29] can also be extended to breast cancer. The modes of BMP2 or BMP6 to initiate their respective pathways are very distinct, as they use different high-affinity type I receptors to initiate signaling [64]. While BMP2 preferentially binds to BMPR1A [65], BMP6 has a higher affinity to ACVR1 [66]. This suggests that BMP-mediated regulation of the canonical WNT pathway may not be specific to one type I receptor. Furthermore, we demonstrated that the BMP-induced decrease in the level of total β-CATENIN protein could be mirrored in the level of nuclear β-CATENIN, and partially contributes to the downregulation of its target gene expression, e.g., *AXIN2*. On the other hand, we also showed that some target genes such as *CCND1* were affected differently by BMP, possibly due to WNT-independent regulation of those target genes. Although *CCND1* and *c-MYC* are considered as target genes of the WNT pathway, due to their crucial roles in the cell cycle and cellular growth, their expression is influenced by several mitogenic signals, including PI3K and ERK pathways [67]. Therefore, degradation of β-CATENIN may trigger the activation of other signaling cascades to compensate for the decrease in *c-MYC* and *CCND1* mRNA expression, and the re-establishment of endogenous gene expression level of related genes could cause the fluctuation in the expression level. Of note, BMP signaling regulates most of those mediators, suggesting that this may occur independently of its effect on β-CATENIN [68]. Supporting this, the decrease in *AXIN2*, which was previously shown as a direct target of β-CATENIN [10], was more robust at the end of 48 h of BMP stimulation compared to others.

Similarly to these findings, BMP stimulation was only able to partially disrupt E-CADHERIN/β-CATENIN junctional complex formation and E-CADHERIN localization, whereas the total protein level of E-CADHERIN remained unchanged, and the migration ability of MCF7 cells was not affected. Bhattacharyya et al. previously showed that inhibition of E-CADHERIN could disrupt localization and target gene expression of β-CATENIN in mouse embryonic stem cells [69]. Therefore, E-CADHERIN may not solely depend on β-CATENIN for the cadherin complex formation, though E-CADHERIN is critical for a proper membrane localization of β-CATENIN. On the other hand, the BMP pathway itself has been shown to stimulate the E-CADHERIN expression in different contexts [70,71]. Therefore, here, BMP signal activation could partially compensate for the disruption of E-CADHERIN junctional localization in a β-CATENIN-independent manner, preventing us from seeing a dramatic change in E-CADHERIN localization. 

Although we found a mechanism for BMP-induced phosphorylation of β-CATENIN in MCF7 and T47D cells, BMP pathway activation in other SMAD4-positive MDA MB 231 cells did not initiate the same mechanism. Likewise, BMP stimulation in SMAD4-negative MDA MB 468 cells caused a stable or rather increasing trend in β-CATENIN protein levels, and exogenous expression of SMAD4 had no further effect on β-CATENIN levels. None of the cell lines we used are listed for β-CATENIN mutation [72]. Additionally, it was previously shown that phosphorylation-induced degradation cascade for β-CATENIN could be triggered in MDA MB 231 cells [73], confirming a functional β-CATENIN phosphorylation and degradation mechanism in this cell line. On the other hand, MCF7 cells have been reported for a gene amplification on the *APC* gene locus, encoding a member of the β-CATENIN destruction complex [74]. In the light of this altered response among breast cancer cell lines, our data suggest that SMAD4 may not be sufficient, and the action of some other critical mediators is needed to trigger the phosphorylation and degradation of β-CATENIN. Among the cell lines we used in this study, MDA MB 231 and MDA MB 468 cells have a ~2-fold higher number of mutations in their genome [74], which could also affect genes encoding for those required co-mediators in these cell lines and could explain their unresponsiveness to BMP-induced β-CATENIN regulation. For instance, overexpression of P53 induces degradation of β-CATENIN through increased phosphorylation at the Ser 33, 37 and Thr41 residues [75], and SMADs have been previously shown for their interaction with P53 to facilitate DNA binding of SMADs for the activation of some of the SMAD target genes [76]. Voorneveld et al. showed that BMP-mediated regulation of the WNT pathway activity may go in both directions depending on the SMAD4 expression and P53 mutation status in colorectal cancer, where they demonstrated that SMAD4 expression negatively correlates with nuclear β-CATENIN in immunohistochemistry samples of patients [77]. Of note, BMP activation could only inhibit the WNT pathway when cells bear wild-type copies of the *TP53* gene [77]. In line, it was also reported that overexpression of oncogenic point mutants of TP53 causes the accumulation of β-CATENIN in human hepatocellular carcinoma cells in vitro [78]. It is worth mentioning here that MCF7 is the only cell line with wild type TP53 status, whereas both MDA MB 231 and MDA MB 468 cells have oncogenic point mutations with R280K and R273H alterations in *TP53*, respectively [79,80], affecting DNA binding ability and impairing P53-induced gene activation. As we observed a similar pattern to the findings of the aforementioned studies, the mutation status of P53 could directly or indirectly affect the expression of co-mediator factors required for β-CATENIN destruction complex to initiate the sequential phosphorylation events upon BMP stimulation. Vice versa, the gain of function mutations of P53 in MDA MB 231 and MDA MB 468 cell lines could prevent the activation of this degradation cascade by interacting with other proteins with gained new functions. Therefore, future studies should address the question whether the mutation status of *TP53* has any impact on the regulation of BMP-induced degradation of β-CATENIN in the context of breast cancer. For instance, a better understanding of the effect of overexpressed mutant *TP53* construct and/or silencing wild-type *TP53* on endogenous β-CATENIN in MCF7 cells would provide more insights into the potential correlation between P53 status and SMAD4 during the BMP-induced regulation of β-CATENIN protein stability. Although the mutation rate in β-CATENIN is relatively less common for breast cancer, *TP53* mutations are frequently observed [81]. Intriguingly, T47D cells also have a point mutation (L194F) on *TP53*. However, the mutations on P53 were shown with the oncogenic gain of function features in the other two cell lines [82,83,84,85], while the mutation has been mainly reported with its loss of function effects in T47D cells [86]. Since we could observe the phosphorylation of β-CATENIN upon BMP stimulation in this cell line, it further implies that rather than wild-type P53, the specific mutant forms of the P53 might cause the advantageous effect to prevent BMP-induced β-CATENIN regulation. Besides the potential contribution of P53 status, these breast cancer cell lines are classified in different molecular subtypes of breast cancer [87]. MCF7 and T47D cells are in the same subgroup, whereas the other two cell lines are listed as triple-negative breast cancer cell lines due to lacking certain receptor expression. Of note, both MCF7 and T47D cells express the estrogen receptor [87]. It was previously reported that β-CATENIN interacts with estrogen receptor-alpha (*ESR1*), and estrogen stimulation increases β-CATENIN protein level in MCF7 cells [88]. Likewise, inhibition of β-CATENIN decreases ESR1 expression in MCF7 cells [89]. However, silencing of *ESR1* expression did not affect the BMP-induced phosphorylation of β-CATENIN in T47D cells, suggesting that ESR1 is not involved in BMP-induced phosphorylation of β-CATENIN. Although we validated that BMP induced phosphorylation of β-CATENIN in MCF7 and T47D cells, phosphorylated β-CATENIN was not degraded in T47D cells. The findings of Aka et al. on the proteomic analysis of MCF7 and T47D cells revealed that there are more than 164 differentially and/or uniquely expressed genes for these cell lines [90]. Of note, some of these differentially expressed proteins are involved in the regulation of protein stability [90]. For instance, HSP70, a chaperone protein regulating protein stability, is among the differentially expressed genes, and its protein level is significantly more abundant in T47D cells than in MCF7 cells [90]. Similarly to the degradation of β-CATENIN, the majority of misfolded proteins are targeted for degradation through the ubiquitin–proteasome-mediated cascade. The chaperones detect those unstable proteins, and may enhance their stability and/or prevent the degradation before initiating the degradation cascade [91,92]. HSP70 has been reported as enhancing the protein stability of oncogenic proteins such as ΔNp63 in the squamous cell carcinoma cell line [93] and β-CATENIN in the murine colorectal cancer cell model [94], respectively. Therefore, the elevated level of HSP70 in T47D cells could be a reason for preventing β-CATENIN from degradation, yet it is phosphorylated.

Lastly, some limitations prevented us from assessing the clinical relevance of our observation in breast cancer patients, although we were able to propose a mechanism with our data. Since BMP-induced regulation of β-CATENIN occurs at the protein level, we could not exploit the TCGA mRNA dataset of the breast cancer patient cohort for the analysis of a potential correlation between BMP pathway activity and the β-CATENIN protein level. On the other hand, Reverse Phase Protein Array (RPPA) data for the breast cancer patient cohort, where it is possible to analyze the change in β-CATENIN protein level, did not include either BMP2/BMP6 or phosphorylated SMADs (SMAD1/SMAD5) or direct target genes of BMP pathway such as ID1–3. Therefore, it was not possible to seek a potential correlation.

Overall, our results suggest that BMP-induced regulation of the canonical WNT pathway could be extended to breast cancer cell lines. Although we proposed the mechanism that BMP2 or BMP6 induces the phosphorylation of β-CATENIN through SMAD4 in MCF7 and T47D cells, comparative experiments in other breast cancer cell lines suggest that this regulation may occur in a cell line-specific manner and that their different genetic background could affect the responsiveness. As we could only observe the phosphorylation of β-CATENIN in two cell lines representing the luminal subtype, it also implies that β-CATENIN could be differentially regulated in different molecular subtypes. Therefore, although our results hold the promise that BMP pathway activity could be exploited for the improvement of WNT inhibitors to prevent breast cancer progression, it has to be first addressed, with follow-up studies, what decides the critical switch from the pro-degradation role to unresponsiveness downstream of BMP pathway activity for the regulation of β-CATENIN protein stability.

## 4. Materials and Methods

### 4.1. Cell Lines and Culture Conditions

MCF7, T47D, MDA MB 468 and MDA MB 231 (ATCC, Manassas, VA, USA) cells were cultured in high-glucose Dulbecco’s modified Eagle’s medium (DMEM) supplemented with a 3.7 g/L sodium bicarbonate (41966029, GIBCO, Paisley, UK), 10% fetal bovine serum (FBS) (S0115, Biochrom, Berlin, Germany) and 1% penicillin/streptomycin (P06-07100, PAN-Biotech, Aidenbach, Germany) antibiotic cocktail. Cells were cultured in a humidified atmosphere containing 5% CO_2_ and 95% air at 37 °C. Cells were passaged every 3–4 days in a 1:4 ratio before reaching the maximum 80% of confluence.

### 4.2. Cell Stimulation and Chemical Inhibition

Cells were seeded at a density of 120,000 cells/well for MCF7, T47D and MDA MB 468 cells, and 90,000 cells/well for MDA MB 231 cells, into a 12-well plate, and they were cultured overnight in the growth medium. The next day, the cells were starved in 1% FBS containing DMEM for 3 h, followed by 24 h of stimulation with 10 nM of BMP2 or BMP6 in normal growth medium. During the LDN193189 (6053, Tocris Bioscience, Bristol, UK) and SB431542 (Sigma) chemical inhibition experiments, they were included in the starvation medium and incubated for 3 h prior to BMP stimulation. Then, cells were stimulated with BMPs for 24 h in the growth medium. An equal amount of DMSO was added to control samples. Recombinant BMP2 was kindly provided by Walter Sebald from Julius-Maximilans-Universität, Würzburg, Germany, and BMP6 was kindly provided by Prof. Slobodan Vukicevic from the University of Zagreb, Croatia.

### 4.3. RNA Isolation and qRT-PCR

Total RNA was isolated by using the NucleoSpin RNA II isolation kit (Macherey-Nagel, Düren, Germany) according to the manufacturer’s instructions and treated with rDNase (MACHEREY-NAGEL, Düren, Germany) to prevent DNA contamination. cDNA was synthesized with MMLV reverse transcriptase from 1 μg total RNA using random hexamer primers (M0253, New England Biolabs, Frankfurt, Germany). PCR amplification and detection were done on StepOne Plus (Applied Biosystems, Singapore) using Luna PCR Master Mix (M3003, New England Biolabs, Frankfurt, Germany) according to the manufacturer’s instructions. GAPDH was used for normalization as a housekeeping gene, and relative mRNA levels were calculated using the ΔΔCT method. All experiments were repeated in at least 3 independent sets. The following primer pairs were used: CCND1 5′-TCTACACCGACAACTCCATCCG-3′, 5′-TCTGGCATTTTGGAGAGGAAGTG-3′; c-MYC 5′-CCTGGTGCTCCATGAGGAGAC-3′, 5′-CAGACTCTGACCTTTTGCCAGG-3′; AXIN2 5′-CAAACTTTCGCCAACCGTGGTTG-3′, 5′-GGTGCAAAGACATAGCCAGAACC-3′; ID1 5′-GCTGCTCTACGACATGAACG-3′, 5′-CCAACTGAAGGTCCCTGATG-3′; ID2 5′-GTGGCTGAATAAGCGGTGTT-3′, 5′-TGTCCTCCTTGTGAAATGGTT-3′; β-CATENIN 5′-CACAAGCAGAGTGCTGAAGGTG-3′, 5′-GATTCCTGAGAGTCCAAAGACAG-3′; GAPDH 5′-GAAGGTGAAGGTCGGAGTC-3′, 5′-GAAGATGGTGATGGGATTTC-3′; β-ACTIN 5′-GGACTTCGAGCAAGAGATGG-3′, 5′-AGCACTGTGTTGGCGTACAG-3′.

### 4.4. Transient Transfections

Cells were seeded at a density of 70,000 cells/well for MDA MB 468 cells into a 12-well plate and were cultured overnight in the growth medium. Cells were transiently transfected using K4^®^ transfection reagent (T080-1.0, Biontex GMBH, Munich, Germany) according to the manufacturer’s instructions. For 1 µg plasmid DNA, 3 µL of K4 reagent was used. Prior to adding the transfection mix, the growth medium was replaced with a 1:1 ratio of growth medium and Opti-MEM™ (51985034, GIBCO, Paisley, UK). Cells were either transfected by pcDNA 3.1 empty backbone vector or human SMAD4 cDNA-containing constructs. Plasmid for the human wild-type SMAD4 expression construct was a kind gift from Prof. Mark de Caestecker, and its generation was previously explained in Shioda et al. (1998) [95]. After 24 h of transfection, cells were starved in 1% FBS containing DMEM for 3 h, and then they were stimulated by 10 nM BMP2 for another 24 h in growth medium. For siRNA-mediated knockdown of SMAD4 and SMAD3, cells were seeded at a density of 90,000 cells/well for MCF7 and T47D cells, and 75,000 cells/well for MDA MB 231 cells, into a 12-well plate. They were cultured overnight in the growth medium. Lipofectamine RNAiMAX (13778075, Invitrogen, Vilnius, Lithuania) was used. Prior to adding the transfection mix, the growth medium was replaced with a 1:1 ratio of growth medium and Opti-MEM™ (51985034, GIBCO, Paisley, UK). Thereafter, 100 nM of siRNA was used per well and mixed with 4 µL of Lipofectamine RNAiMAX and incubated overnight. The next day, cells were starved in 1% FBS containing DMEM for 3 h, and then they were stimulated by 10 nM of BMP2 for another 24 h in growth medium. All siRNAs were obtained from Dharmacon-Healthcare (Lafayette, CO, USA). ON-TARGET plus non-targeting siRNA (si-scr) was used as control 5′-UGGUUUACAUGUCGA CUAA-3′ (D-001810-01). Human SMAD4 siRNA #1 5′-GUACAGAGUUACUACUUAG-3′ (J-003902-12-0002) and SMAD4 siRNA #2 5′-GCAAUUGAAAGUUUGGUAA-3′ (J-003902-09-0002) were used to deplete SMAD4. Human SMAD3 siRNA 5′-GAGUUCGCCUUCAAUAUGA-3′ (J-020067-06-0002) was used to deplete SMAD3.

### 4.5. Western Blotting

Protein lysates were separated by 10% SDS–PAGE and subsequently transferred on polyvinylidene difluoride (PVDF) membranes at 100 V for 2 h in the cold room. Membranes were blocked for 1 h with 5% bovine serum albumin in TBS-T, and were incubated with primary antibody dilutions overnight at 4 °C. The next day, membranes were incubated with respective HRP-conjugated secondary antibodies for 1 h and analyzed using WesternBright Quantum ECL HRP reagents (K-12045, Advansta, San Jose, CA, USA) on Fusion-FX7 detection system by using FusionCapt Advance Solo4 software (16.13a) (Vilber-Lourmat, Marne-la-Vallée, France). The following primary antibodies were used: anti-pSMAD1/5/9 (13820), anti-β-CATENIN (9581), anti-p-GSK3ß (9323S), p-β-CATENIN (9561S), anti-GAPDH (2118S), anti-p-SMAD3 (9520S) and anti-SMAD3 (9523S) from Cell Signaling Technologies (Leiden, The Netherlands), anti-SMAD4(sc-7966) from Santa Cruz (Dallas, TX, USA), anti-LAMINB1 (5G8-D3-H7) from Biolegend (Koblenz, Germany) and anti-ß-ACTIN (A5441) from Sigma Aldrich (St. Louis, MO, USA).

### 4.6. Cellular Fractionation Assay

A total of 1.5 × 10^6^ of MCF7 cells were seeded into the 10 cm plate for each condition prior to the cell fractionation assay. After overnight incubation, cells were starved for 3 h in 1% FBS containing DMEM, and then were stimulated by 10 nM of BMP2 for 48 h in growth medium. First, cells were scraped in ice-cold phosphate-buffered saline and harvested by centrifugation. Fractionation was done by using Cell Fractionation Kit-Standard (ab109719, Cambridge, UK) according to the manufacturer’s instructions. Fractions were separated by SDS-PAGE and analyzed by immunoblotting. LaminB1 and GAPDH were used as positive and negative controls for nuclear fraction, respectively.

### 4.7. Immunostaining and Confocal Microscopy

MCF7 cells were fixed with 4% PFA, permeabilized with 0.3% Triton X-100 (37240, Serva, Heidelberg, Germany) for 10 min, and subsequently blocked with 5% bovine serum albumin/5% normal goat serum solution before incubation with specific primary antibodies. The following antibodies were used: anti-β-CATENIN (9581) (1:200), anti-E-CADHERIN (14472S) (1:250) and anti-pSMAD1/5/9 (13820) (1:250) from Cell Signaling Technologies (Leiden, The Netherlands). Samples were incubated at 4 °C overnight followed by 1 h of Alexa Fluor 488 or 594–conjugated secondary antibody (1:400) (Invitrogen) incubation. Nuclei were stained with 4′,6-diamidino-2-phenylindole (DAPI) (1:1000), and the cytoskeleton was stained with Phalloidin CruzFluor™-647 (sc-363797, Dallas, TX, USA) (1:500). Images were taken with an inverted confocal SP8 microscope (Leica, Wetzlar, Germany) from at least three random positions and analyzed with ImageJ (v 1.54). The semi-automated Junction Mapper macro was used to quantify junctional β-CATENIN and E-CADHERIN immunofluorescence staining [57]. β-CATENIN staining was used as a template to decide on the junctions’ frames. At least 10 cells from each image of control and BMP2-stimulated staining were analyzed. The total fluorescence intensity of the junctions’ staining was divided by the respective length of the junctions. The mean of the fluorescence intensities of BMP2-stimulated samples were normalized to control samples. Three different sets of experiments were represented in the graphs.

### 4.8. Wound Healing Migration Assay

Cells were seeded on a 6-well plate at a density of 300,000 cells/well and cultured overnight. Then, cells were transfected with scrambled or SMAD4 targeting siRNAs by using Lipofectamine RNAiMAX (Invitrogen, Vilnius, Lithuania). At 24 h post-transfection, cells were starved for 3 h and were incubated with BMP2 (10 nM) for another 24 h. Then, cells were re-seeded into plastic migration inserts (80209, Ibidi GMBH, Gräfelfing, Germany) at a density of 50,000 cells/well. When they reached confluency, inserts were removed for the migration assay. Prior to initiation of the assay, cells were treated with Mitomycin C (10 μg/μL) (S8146, Sellekchem, Cologne, Germany) for 2 h to inhibit cell proliferation.

### 4.9. Co-Immunoprecipitation Assay

MCF7 and MDA-MB-231 cells were seeded at a density of 1.5 × 10^6^ cells/plate on 10 cm plates. Two days after seeding, cells were washed with cold PBS and lysed with the RIPA lysis buffer (150 mM NaCl, 25 mM Tris/HCl, 0.1% SDS, 0.5% NP-40 [pH 7.8]) supplemented with inhibitors (1 mM PMSF, 2 mM Na_3_VO_4_, 20 mM Na_4_P_2_O_7_, 50 mM NaF, complete protease inhibitor cocktails [11697498001, Roche, Mannheim, Germany]). Cell lysates were centrifuged, and supernatants were transferred to new tubes. The total cell lysate for each sample was separated into new tubes and denatured by adding Laemmli sample buffer. Supernatants were incubated with either IgG isotype control (211-032-071, Dianova, Hamburg, Germany) or SMAD3 antibody on a wheel rotator at 4 °C overnight. Immunocomplexes were precipitated with protein A-Sepharose beads (17127901, GE Healthcare, Uppsala, Sweden) at 4 °C for 2 h. Beads were washed 5 times with an inhibitor-supplemented lysis buffer. Laemmli sample buffer was added to samples and boiled for 5 min at 95 °C, and Western blotting was performed to detect the co-immunoprecipitation of β-CATENIN with SMAD3. The experiment was done once for each cell line.

### 4.10. Statistical Analysis

For statistical analysis, an unpaired Student’s *t*-test with Welch’s correction or one-way analysis of variance (ANOVA) with post-hoc Tukey HSD test was used, depending on the experiment (unless otherwise stated); *p* < 0.05 was considered as statistically significant.

## Data Availability

TCGA datasets used for differential ligand expression in breast cancer patients were retrieved from the GEPIA2 online analysis tool. All settings were used as default and could be accessed via GEPIA2.

## Supplementary Materials

The following supporting information can be downloaded at: https://www.mdpi.com/article/10.3390/ijms25094593/s1.
