# Supplementary material for: BMP Stimulation Differentially Affects Phosphorylation and Protein Stability of β-Catenin in Breast Cancer Cell Lines
Source: Int J Mol Sci. 2024 Apr 23;25(9):4593. doi: 10.3390/ijms25094593 (PMC11083028; doi:10.3390/ijms25094593)
Supplement: Supplementary file 1 [file ijms-25-04593-s001.zip › ijms-2902145-supplementary.pdf]

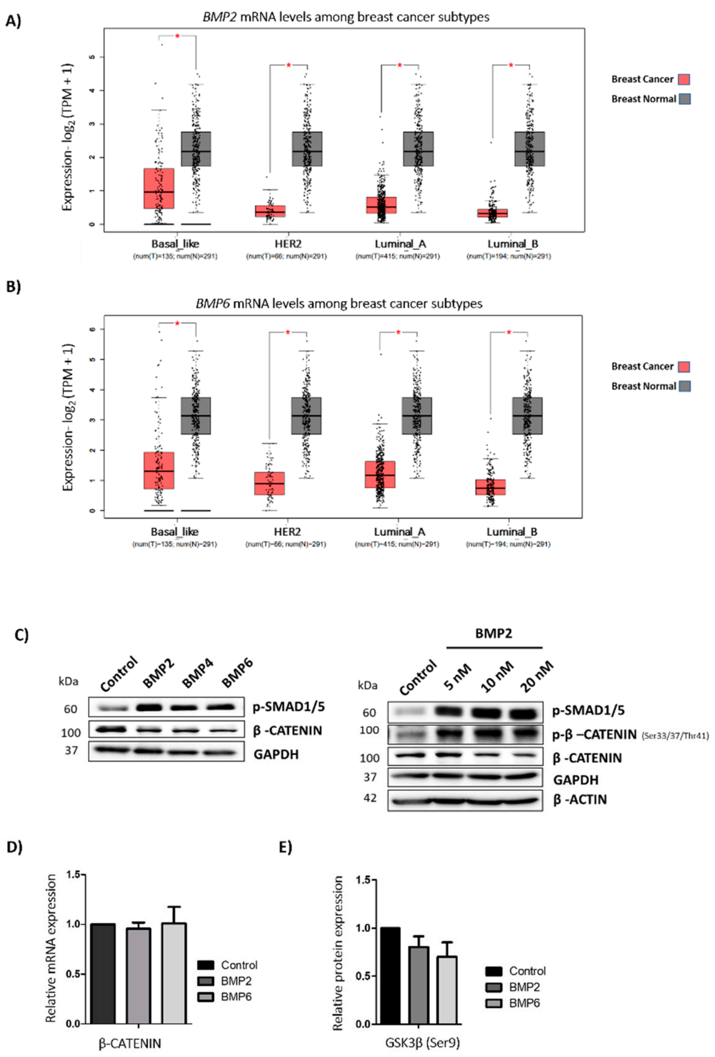

Supplementary Figure S1: BMP stimulation triggers phosphorylation and proteasomal degradation of β-catenin in MCF7 cells. Analysis of TCGA breast tissue sample data. Comparison of mRNA expression levels of (A) *BMP2* and (B) *BMP6* ligands among data from tumor tissue representing different molecular subtypes (#of samples was indicated below the figures) and their comparison to the available data from TCGA normal tissue and Genotype-Tissue Expression (GTEx) (n=291) in the GEPIA2 database. The method for differential analysis is one-way ANOVA, using disease state (Tumor or Normal) as variable for calculating differential expression ($p < 0.01$). Default settings were used, and analysis was retrieved by GEPIA2 web server (Tang, Z. et al. ,2017). (C) Expanded western blot figure showing protein levels of phosphorylated SMAD1/5, total and phosphorylated β-CATENIN upon BMP stimulation (BMP2, BMP4 and BMP6 = 10 nM) (left panel) and dose dependent change in phosphorylated and total β-CATENIN levels upon 24 hours of BMP2 stimulation in MCF7 cells. (D) The effect of BMP2 or BMP6 (10 nM) stimulation after 24 hours on mRNA expression level of β-CATENIN in MCF7 cells, which was analyzed by using qPCR and shown as change in relative mRNA level. (E) Densitometric analysis of the inactive protein level of GSK3β, which is phosphorylated at Ser9 residue upon BMP stimulation. Samples were normalized to GAPDH. Data is represented as the mean + SD of three independent experiments.

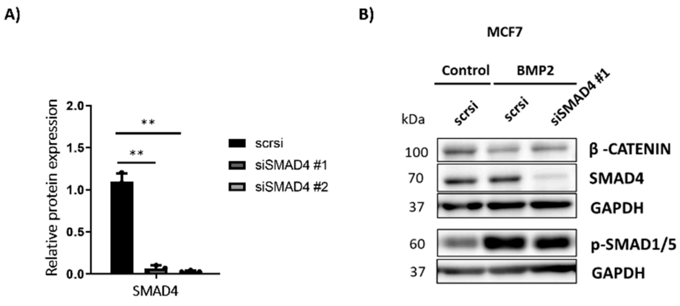

Supplementary Figure S2: BMP-induced degradation of β-CATENIN requires SMAD4 in MCF7 cells (A) Densitometric analysis of SMAD4 protein levels in total cell lysates confirming the efficiency of SMAD4 knock down after siRNA-mediated silencing with two different oligo nucleotides. After transfection with respective oligonucleotides, cells were cultured for 48 hours before harvesting. GAPDH was used as equal loading control, and all values were normalized to GAPDH prior to relative protein expression analysis. Data is represented as the mean + SD of three independent experiments (**$p < 0.01$). (B) Representative western blot image showing the change in protein levels of total β-CATENIN, SMAD4, and phosphorylated SMAD1/5 in MCF7 cells after silencing with other SMAD4 siRNA. GAPDH was shown as an equal loading control protein. In SMAD4 experiments, siSMAD4#2 was used for gene silencing in main figures.

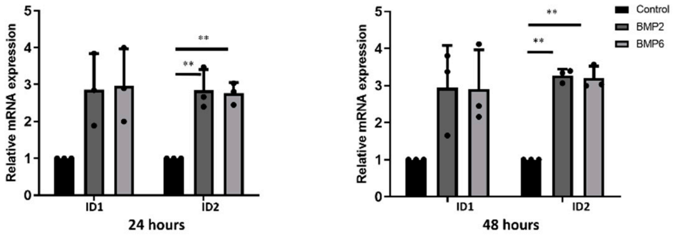

Supplementary Figure S3: Relative mRNA expressions of BMP target genes upon BMP stimulation. ID1 and ID2 were analyzed by qRT-PCR after 24 hours or 48 hours of BMP2 or BMP6 stimulation in MCF7 cells. Cells were starved for 3 hours and followed by BMP stimulation for respective time points. All targets were normalized to GAPDH as a housekeeping gene prior to relative expression quantification. Data is represented as the mean + SD of three independent experiments (*$p < 0.05$, **$p < 0.01$).

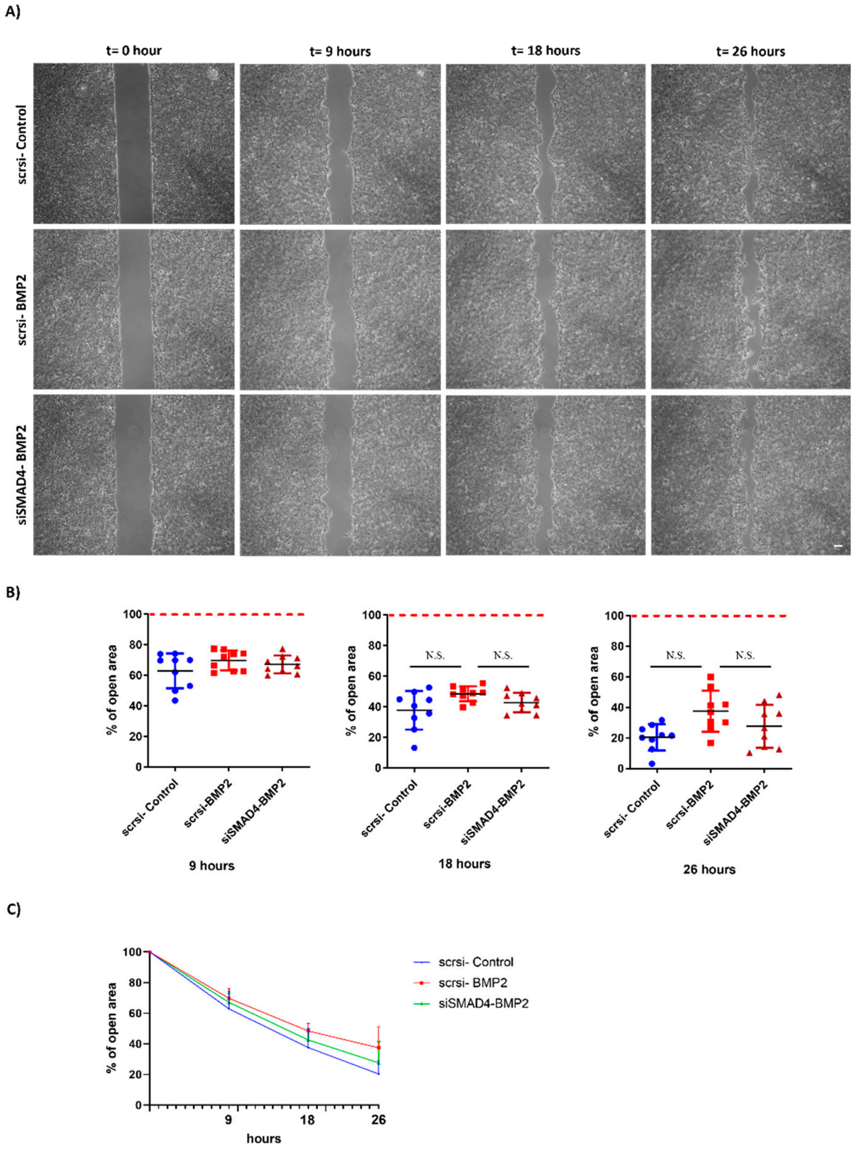

Supplementary Figure S4: BMP-induced degradation of β-CATENIN did not affect migration ability of MCF7 cells. (A) Representative images of wound healing migration assay showing the open wound area after respective time points in MCF7 cells. Cells were transfected with scrambled (scr) or SMAD4 siRNA oligonucleotides and 24 hours of post-transfection, cells were incubated with BMP2 (10 nM) for another 24 hours. Then, cells were seeded into plastic migration inserts. When they reached confluency, inserts were removed for the migration assay. Prior to initiation of assay, cells were treated with Mitomycin C (10 μg/μl) for 2 hours to inhibit cell proliferation. (B-C) Quantification of open wound area percentage at 9 hours, 18 hours, and 26 hours time points of the migration assay in MCF7 cells, respectively. Data is represented as the mean + SD of at least three random positions from each of the three independent experiments. (N.S: not significant).

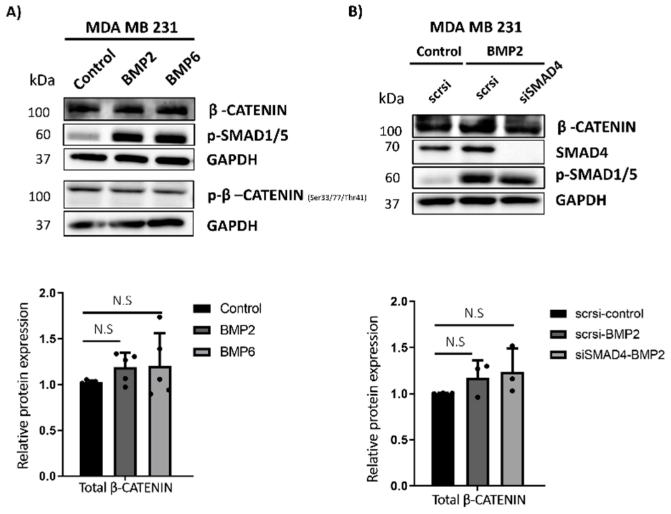

Supplementary Figure S5. BMP stimulation differentially affects β-CATENIN protein stability in MDA MB 231 cells. (A) Representative western blot images showing total β-CATENIN, phosphorylated SMAD1/5 and densitometric analysis of total β-CATENIN in MDA MB 231 cells after BMP2 or BMP6 stimulation (10 nM). Cells were starved for 3 hours and stimulated with respective BMPs for 24 hours. (B) Representative western blot images of total β-CATENIN, SMAD4, phosphorylated SMAD1/5 and densitometric analysis of total β-CATENIN in MDA MB 231 cells. After silencing SMAD4, cells were starved for 3 hours and stimulated for an additional 24 hours with BMP2 (10 nM). GAPDH was used as equal loading control, and all values were normalized to loading control prior to relative protein expression analysis. Data is represented as the mean + SD of at least three independent experiments. (N.S: not significant).

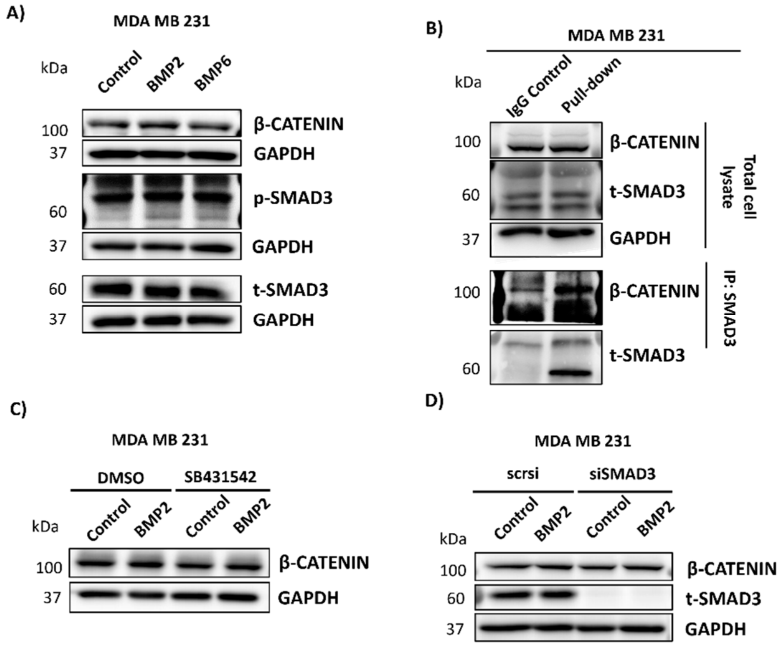

Supplementary Figure S6: (A) Representative western blot images of three independent experiments showing the level of β-CATENIN, phosphorylated and total SMAD3 upon BMP2 and BMP6 stimulation (10 nM for each) in MDA MB 231 cells. Cells were starved for 3 hours and followed by 24 hours of BMP stimulation. (B) Co-immunoprecipitation assay showing the interaction between SMAD3 and β-CATENIN in MDA MB 231 cells. (C) Representative western blot images showing the level of total β-CATENIN after SB431542-induced chemical inhibition of TGF-β pathway activity in the absence or presence of BMP2 stimulation. During starvation, cells were treated with 10 μM SB431542 or equal volume of DMSO for 3 hours. Then, cells were stimulated by 10 nM BMP2 for 24 hours. (D) Representative western blot images showing the level of total β-CATENIN and total SMAD3 proteins after siRNA-mediated silencing of SMAD3 in MDA MB 231 cells. After 24 hours of transfection, cells were starved for 3 hours and stimulated for an additional 24 hours with BMP2 (10 nM). GAPDH was used as an equal loading control. Chemical inhibition and siRNA-mediated silencing of SMAD3 experiments were shown as a representative figure of two independent experiments.
